# Targeting the Bacterial Cytoskeleton of the *Burkholderia cepacia* Complex for Antimicrobial Development: A Cautionary Tale

**DOI:** 10.3390/ijms19061604

**Published:** 2018-05-30

**Authors:** Sonya C. Carnell, John D. Perry, Lee Borthwick, Daniela Vollmer, Jacob Biboy, Marcella Facchini, Alessandra Bragonzi, Alba Silipo, Annette C. Vergunst, Waldemar Vollmer, Anjam C. M. Khan, Anthony De Soyza

**Affiliations:** 1 Institute of Cellular Medicine, Newcastle University, Newcastle NE2 4HH, UK; sonya.carnell@ncl.ac.uk (S.C.C.); lee.borthwick@ncl.ac.uk (L.B.); 2 Department of Microbiology, The Freeman Hospital, Newcastle NE7 7DN, UK; john.perry@nuth.nhs.uk; 3 Centre for Bacterial Cell Biology, Institute for Cell and Molecular Biosciences, Newcastle University, Newcastle NE2 4HH, UK; daniela.vollmer@ncl.ac.uk (D.V.); Jacob.Biboy@newcastle.ac.uk (J.B.); waldemar.vollmer@newcastle.ac.uk (W.V.); anjam.khan@newcastle.ac.uk (A.C.M.K.); 4 Infections & Cystic Fibrosis Unit, San Raffaele Scientific Institute, 20132 Milan, Italy; facchini.marcella@hsr.it (M.F.); bragonzi.alessandra@hsr.it (A.B.); 5 Department of Chemical Sciences, University of Naples Federico II, Via Cintia 4, 80026 Napoli, Italy; silipo@unina.it; 6 VBMI, INSERM, Univ. Montpellier, 30908 Nimes, France; annette.vergunst@univ-montp1.fr

**Keywords:** antimicrobial, cytoskeleton, Burkholderia

## Abstract

*Burkholderia cepacia* complex (BCC) bacteria are a group of opportunistic pathogens that cause severe lung infections in cystic fibrosis (CF). Treatment of BCC infections is difficult, due to the inherent and acquired multidrug resistance of BCC. There is a pressing need to find new bacterial targets for antimicrobials. Here, we demonstrate that the novel compound Q22, which is related to the bacterial cytoskeleton destabilising compound A22, can reduce the growth rate and inhibit growth of BCC bacteria. We further analysed the phenotypic effects of Q22 treatment on BCC virulence traits, to assess its feasibility as an antimicrobial. BCC bacteria were grown in the presence of Q22 with a broad phenotypic analysis, including resistance to H_2_O_2_-induced oxidative stress, changes in the inflammatory potential of cell surface components, and in-vivo drug toxicity studies. The influence of the Q22 treatment on inflammatory potential was measured by monitoring the cytokine responses of BCC whole cell lysates, purified lipopolysaccharide, and purified peptidoglycan extracted from bacterial cultures grown in the presence or absence of Q22 in differentiated THP-1 cells. BCC bacteria grown in the presence of Q22 displayed varying levels of resistance to H_2_O_2_-induced oxidative stress, with some strains showing increased resistance after treatment. There was strain-to-strain variation in the pro-inflammatory ability of bacterial lysates to elicit TNFα and IL-1β from human myeloid cells. Despite minimal toxicity previously shown in vitro with primary CF cell lines, in-vivo studies demonstrated Q22 toxicity in both zebrafish and mouse infection models. In summary, destabilisation of the bacterial cytoskeleton in BCC, using compounds such as Q22, led to increased virulence-related traits in vitro. These changes appear to vary depending on strain and BCC species. Future development of antimicrobials targeting the BCC bacterial cytoskeleton may be hampered if such effects translate into the in-vivo environment of the CF infection.

## 1. Introduction

The *Burkholderia cepacia* complex (BCC) constitutes a group of over 20 closely related opportunistic respiratory pathogen species associated with life-threatening infections in cystic fibrosis (CF) and other immunocompromised patients. Of these species, *B. cenocepacia* and *B. multivorans* are the most prevalent causes of infections in CF patients, and some strains have proved to be highly transmissible between CF patients. BCC infections may remain established for many months, or even years, with clinical outcomes highly variable and unpredictable; in some cases, they can lead to a severe and often fatal complication known as “cepacia syndrome”. Cepacia syndrome is characterised by a rapid clinical decline, with high fevers and bacteraemia, progressing to severe pneumonia and death; its existence has led to BCC bacteria emerging as important respiratory pathogens within the CF community. Their intrinsic and acquired resistance to most clinically relevant antimicrobials makes BCC infections notoriously difficult to eradicate or manage. Understandably, due to concerns over this multidrug resistance in BCC and other respiratory pathogens, such as *Pseudomonas aeruginosa*, the development of new and novel antimicrobials is urgently required.

The majority of clinically available antibiotics generally target synthetic pathways, such as DNA synthesis, protein synthesis, and RNA synthesis (reviewed in [1]), suggesting that there is a need to identify new bacterial therapeutic targets that have an important cellular function or role. A number of groups have revealed the presence of the bacterial cytoskeleton, and its potential as an antimicrobial target has been recognized [2]. One cytoskeletal protein of particular interest is the widely conserved actin homolog, MreB. This plays a role in a number of cellular functions, including maintaining cell shape in rod-like bacteria. More recently the role of MreB has been found to be associated with cell-surface-located virulence factors, further emphasizing the suitability of this protein as an antimicrobial target. In the case of *P. aeruginosa*, MreB is required for motility and cell surface localisation of the type IV pilus, a cell surface adhesin important for adhesion to mammalian cells during infection and biofilm formation [3]. Additionally, Bulmer and colleagues have demonstrated the MreB-associated cytoskeletal protein MreC is required for expression of *Salmonella* Typhimurium type three secretion system-1 (T3SS-1) and flagella complexes, both important pathogenicity factors required for colonisation and invasion of the intestine in vivo [4].

To date, few inhibitors of MreB have been identified. *S*-(3,4-dichlorobenzyl) isothiourea hydrochloride (A22) is a small molecule inhibitor of MreB, originally identified as an inhibitor of replication in *E. coli* [5]. A22 has an antimicrobial effect on a range of Gram-negative bacteria; however, very little is known about the effects of A22 on virulence factor expression. Studies of *Shigella flexneri* have shown that exposure to sub-MIC levels of A22 can reduce its ability to invade CHO-K1 cells in vitro [6]. A further inhibitor of MreB, CBR-4830, was identified via a whole-cell antibacterial screen for growth inhibitors of efflux-compromised *P. aeruginosa* strains [7]. CBR-4830 is a novel indole MreB inhibitor chemically distinct from A22.

Recently, we assessed the antimicrobial activity of a panel of A22-related isothiourea hydrochloride derivatives against multi-drug resistant clinical isolates of *P. aeruginosa* and BCC [8]. Here, we extend the use of the lead candidate from this panel, *S*-(4-chlorobenzyl) isothiourea hydrochloride, named Q22, a putative MreB inhibitor (also described as C2 by Nicholson et al.), and study the effects of this compound on virulence-related traits of BCC bacteria [8]. We show that sub-lethal treatment of BCC with Q22 altered virulence phenotypes, including increased resistance to oxidative stress and pro-inflammatory potential, suggesting caution should be taken when targeting the bacterial cytoskeleton of BCC for antimicrobial development.

## 2. Results

### 2.1. Q22 Treatment Alters Cell Morphology, Reduces Growth Rate, and Inhibits Growth of B. cenocepacia Species

**Figure 1:**
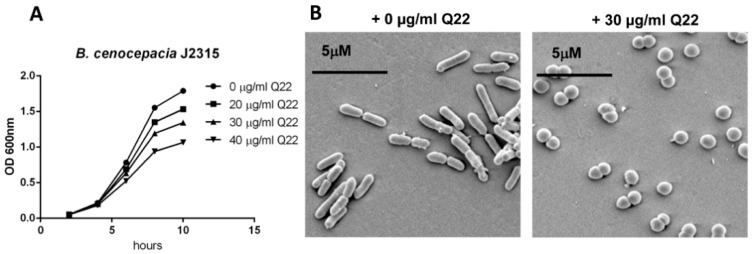
Growth and morphological changes induced by Q22 treatment. (**A**) Growth of *B. cenocepacia* J2315 in the absence or presence of increasing concentrations of Q22 (0, 20, 30, 40 μg/mL); (**B**) scanning electron microscope images of *B. cenocepacia* J2315, untreated and after treatment with 30 μg/mL Q22 at 6 h timepoint.

Having recently established the antimicrobial activity of both A22 and Q22 against a panel of BCC strains [8], we wanted to further analyse the effect of Q22 on growth and morphology of a panel of BCC bacteria (Table 1). Initially we used *B. cenocepacia* J2315 as a test organism to determine appropriate growth permissive levels of Q22 (Figure 1A). *B. cenocepacia* J2315 is a clinical ET-12 epidemic strain isolated from a CF patient [9]. Q22-treated *B. cenocepacia* J2315 cultures showed a reduction in growth rate in a concentration-dependent manner (Figure 1A; 0 μg/mL vs. 40 μg/mL Q22; *p* ≤ 0.05)). Based on these results, 30 μg/mL Q22 was selected as an appropriate, sub-lethal, growth-permissive concentration for subsequent experiments. Upon exposure to 30 μg/mL Q22, all BCC strains tested, including those listed in Table 1, demonstrated a reduction in growth rate (as previously [8]). The degree of growth rate reduction varied between strains, and a lesser reduction was observed upon exposure to Q22 when compared to A22. Changes in cell morphology from rod to cocci forms were confirmed by scanning electron microscopy, supporting disruption of the MreB-based cytoskeleton (Figure 1B).

### 2.2. Q22 Treatment Alters Ability of B. cenocepacia to Resist H_2_O_2_-Induced Oxidative Stress

**Figure 2:**
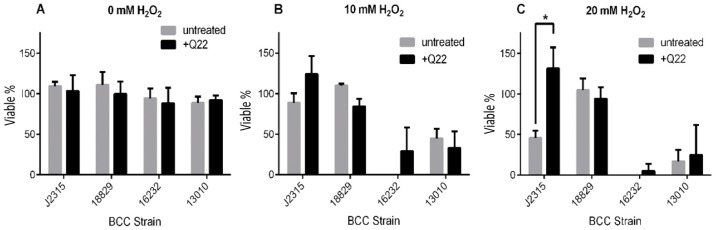
Survival of *B. cepacia* complex (BCC) strains exposed to H_2_O_2_ in vitro. Late-stationary-phase cells were treated with varying concentrations of H_2_O_2_, 0 mM (**A**), 10 mM (**B**), and 20 mM (**C**), as described under Section 4. Samples were plated in triplicate for colony counts, and percentage survival (expressed as % viability) was calculated relative to colony counts of untreated bacteria. * *p* < 0.05. Isolates included *B. cenocepacia* LMG16656 (J2315), *B. cenocepacia* LMG18829, *B. multivorans* LMG13010, and *B. vietnamiensis* LMG16232 (see Table 1).

BCC bacteria are exposed to reactive oxygen species (ROS) during colonisation and infection of the CF lung, and have a number of strategies to combat this form of stress [10,11]. We evaluated the susceptibility of Q22-treated and untreated BCC cultures to H_2_O_2_ (Figure 2). The reduction in viability upon H_2_O_2_ exposure varied between BCC strains when grown without drug treatment, with *B. cenocepacia* strains LMG18829 and J2315 showing the greatest resistance at 20 mM H_2_O_2_ (Figure 2B,C).

When compared to untreated controls, we observed an increase in H_2_O_2_ resistance for strain J2315 that was statistically significant (Figure 2C, *p* < 0.05).

### 2.3. Q22 Treatment Does Not Alter Lipopolysaccharide Profile of B. cenocepacia Strains

We analysed the crude LPS profiles of WCLs prepared from BCC cultures exposed to Q22, to determine if this compound alters LPS composition when compared to untreated WCLs. WCLs were prepared from BCC strains grown in the presence and absence of 30 μg/mL Q22. Of the four strains tested, no significant differences in the LPS electrophoretic profile were observed. J2315 showed no difference in profile at all. To further assess if there were any changes in LPS structure, LPS was isolated from Q22-treated and untreated cultures of *B. cenocepacia* LMG18829, and lipid A extracted and analysed further by MALDI-TOF mass spectrometry (Table 2). Both LPS and lipid A showed a mixture of differently-acylated species, both carrying Ara4N residues on the polar heads. Interestingly, the Q22-treated LMG18829 strain also carried an unsubstituted tetra-acylated lipid A species, absent in the untreated strain (Table 2). However, no other significant differences in LPS structure were observed.

### 2.4. Q22 Treatment Alters Proinflammatory Potential of BCC Strains

**Figure 3:**
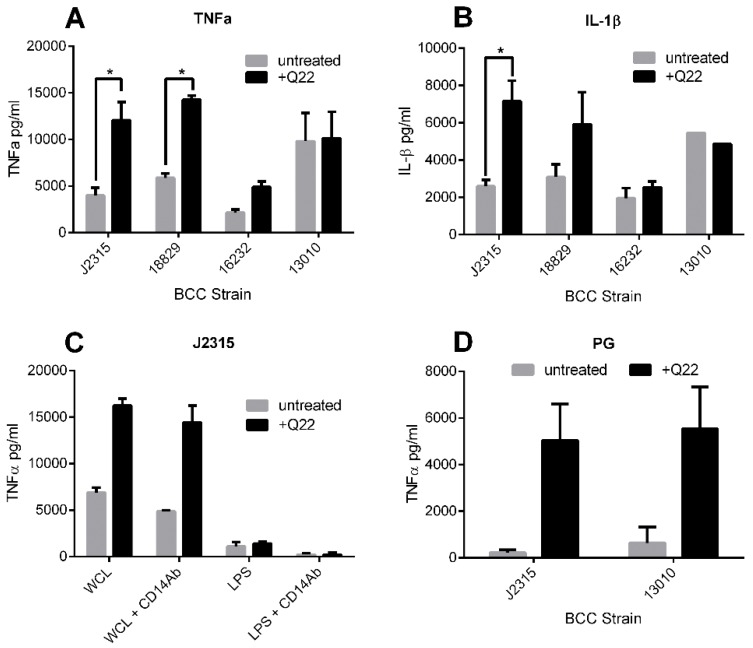
Cytokine responses of THP-1 cells, stimulated with Q22-treated and untreated preparations. Cytokine responses at 6 h post-stimulation with whole cell lysates of BCC strains *B. cenocepacia* J2315, *B. cenocepacia* LMG18829, *B. multivorans* LMG13010, and *B. vietnamiensis* LMG16232. (**A**) TNFα; (**B**) IL-1β. TNFα responses at 6 h post stimulation with whole cell lysates, purified lipopolysaccharide (LPS) (**C**), or purified peptidoglycan (PG) (**D**) from BCC strain *B. cenocepacia* J2315. Comparison of profiles from cells stimulated with and without a CD14 antibody pre-incubation step (**C**). * *p* < 0.05.

BCC LPS has potent endotoxic activity and can elicit high levels of pro-inflammatory cytokines [12,13]. To determine if chemical disruption of the bacterial cytoskeleton using compound Q22 alters the endotoxic potential of the bacteria, we stimulated differentiated THP-1 cells with WCLs, known to be rich in LPS, prepared from cultures exposed to Q22. Pro-inflammatory cytokine responses characteristic of those encountered within the host during BCC infection (TNFα and IL-1β) were measured. Data demonstrated a variable strain-dependent increase in cytokine induction when cells were stimulated with lysates obtained from bacteria exposed to Q22, compared to those obtained from untreated bacteria. This significant strain-specific increase was noted for both TNFα (Figure 3A; *p* < 0.01) and IL-1β (Figure 3B; *p* < 0.05) responses for Q22-treated *B. cenocepacia* strain J2315, as well as for TNFα (Figure 3A; *p* < 0.01) for Q22-treated *B. cenocepacia* strain LMG18829.

To determine whether this strain-specific increase in pro-inflammatory response was LPS-dependent, we pre-incubated THP-1 cells with a CD14 antibody prior to stimulation, to block the TLR4-dependent stimulatory pathway used by LPS. The increased TNFα response observed with *B. cenocepacia* J2315 Q22-treated lysates was not abolished with the CD14 antibody treatment, suggesting the increased cytokine release was not LPS-driven (Figure 3C). This was further confirmed by observing the TNFα response of THP-1 cells, stimulated with purified LPS extracted from *B. cenocepacia* J2315 cultures grown in the presence and absence of Q22. Results demonstrated no significant difference in TNFα response, obtained by stimulation with *B. cenocepacia* J2315 LPS purified from treated and untreated cultures (Figure 3C). This is in agreement with our finding that the LPS profile of *B. cenocepacia* J2315 was not changed after Q22 treatment.

Peptidoglycan (PG) is a significant structural and immune-stimulatory component of the bacterial cell wall, and could plausibly be affected by cytoskeletal disruption. To determine if the observed increase in pro-inflammatory potential of the *B. cenocepacia* WCLs could be attributed to PG, we isolated PG from two different BCC species, *B. cenocepacia* J2315 and *B. multivorans* LMG13010, each grown in the presence and absence of 30 µg/mL Q22. The purified PG was subsequently used to stimulate THP-1 cells, and TNFα responses were measured. Both BCC strains tested showed an increase in TNFα production when the bacteria were exposed to 30 µg/mL Q22 prior to PG extraction, when compared to untreated controls; however, only the increase between the treated and untreated *B. cenocepacia* J2315 samples was statistically significant (*p* < 0.05) (Figure 3D).

### 2.5. Q22 Toxicity In-Vivo Studies in Zebrafish and Mouse Models

Preliminary experiments demonstrated negligible toxicity in THP-1 cells or CF primary bronchial epithelial cells (data not shown) [14].

Ultimately, the potential for in-vivo toxicity must be considered when developing a compound for antimicrobial use; therefore, we aimed to determine the effects of Q22 in vivo, utilising the zebrafish embryo BCC infection model established by Vergunst and colleagues [15], and the BCC mouse lung infection model optimised by Bragonzi and co-workers [16]. We aimed to extend the in vivo toxicity testing in these models prior to Q22 infection protection assessment.

**Figure 4:**
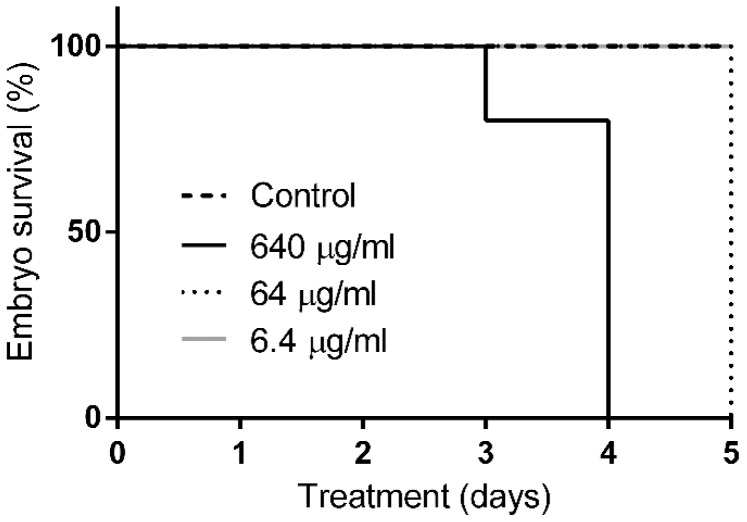
Toxicity of Q22 in the zebrafish embryo model. Zebrafish embryos were exposed to increasing concentrations of Q22 (6.4 µg/mL; 64 µg/mL; 640 µg/mL) at 30 high power field and monitored for survival for up to 120 h post-incubation. Data expressed as percentage survival.

Zebrafish embryos, 30 h post-fertilisation, were incubated in different concentrations of Q22 in embryo water. Whereas 100% of untreated control embryos and embryos incubated in 6.4 µg/mL Q22 survived during a five-day observation period, a dose-dependent killing was observed with higher doses of Q22 than were anticipated to be needed for antimicrobial effects (Figure 4; *p* < 0.0001). A concentration of 64 µg/mL was lethal for the embryos on day five, but embryos started to die on day three when incubated in embryo water with 640 µg/mL, a concentration that killed most of the embryos on day four of the treatment. These results clearly indicate the toxicity of the compound for zebrafish larvae, even at concentrations that are close to the appropriate bacterial, sub-lethal, growth-permissive concentration of 30 µg/mL.

Results of the preliminary toxicity study in mice demonstrated that mice exposed to higher doses of Q22 100 mg/kg (Group 1) and 50 mg/kg (Group 2) after IT administration of Q22 were moribund (could not right themselves after being placed in lateral recumbence), and thus were killed and the experiment terminated (Table 3). Those mice exposed to the lower dose, 25 mg/kg Q22 (Group 3), demonstrated reduced mobility when compared with control mice during the first two days of treatment, and lungs showed signs of inflammation and damage macroscopically after four days. All control mice were healthy after treatment (Group 4). Based on these results suggesting significant pulmonary toxicity, and in the interests of animal welfare, no further in-vivo experiments were performed.

## 3. Discussion

Although the emergence of drug-resistant bacteria is a pressing issue, there are few antimicrobials in development for the treatment of infections caused by these organisms [17]. We assessed the bacterial cytoskeleton as a potential novel antimicrobial target, using the highly antibiotic-resistant BCC as a model group of organisms. To target the bacterial cytoskeleton, we tested the A22-related compound *S*-(4-chlorobenzyl) isothiourea hydrochloride (named Q22 in this study) as a potential antimicrobial candidate, and measured phenotypic changes in the virulence traits of BCC. In addition to the expected changes in growth and morphology observed with other Gram-negative bacterial species [4,18,19], we also observed unexpected increases in resistance to H_2_O_2_-induced oxidative stress and the pro-inflammatory potential of Q22 treatment in selected strains. BCC strains are known to show a heterogeneity with other phenotypes, even across apparently clonal isolates [20]. Hence, the variation herein appears consistent with these prior data.

We are unable to provide a mechanistic explanation for this increased resistance to H_2_O_2_-induced stress in strain J2315. It is possible that the physiological changes in cell shape and structure may alter the release or access to oxidative stress-related enzymes, such as catalases, which may vary between strains. The concept of a drug treatment providing an opportunistic pathogen with the increased capacity to combat ROS is a concern. This is highly relevant, as protection against ROS, such as hydrogen peroxide, is important for the survival of BCC persister cells [21]. Persister cells are cells that have entered a dormant, multidrug-tolerant state, and are thought to play a role in the recalcitrance of biofilm-related infections in vivo.

Furthermore, in-vitro data generated during this study has suggested that Q22 treatment can increase the pro-inflammatory potential of certain BCC. As Q22 has been shown to depolymerise MreB in other Gram-negative organisms [22], it is possible that by depolymerising the bacterial cytoskeleton of BCC, Q22 may alter the structure of important bacterial pathogen-associated molecular patterns (PAMPs) located within the cell wall. Our data suggests that Q22 does not significantly affect the structure of the LPS component of the outer membrane, but may have an effect on the PG component. To confirm a role for PG in the changes in cytokine response of Q22-treated strains, we are currently investigating the structure of PG extracted from Q22-treated bacteria.

Recently, Q22 has been described as a breakdown product of an antibacterial compound, MAC13243, found to inhibit the bacterial lipoprotein targeting chaperone LolA, identified during a chemical genome screen [23]. Saturation transfer difference (STD) NMR analysis revealed that Q22 directly interacts with LolA in vitro. Therefore, we must consider that potential interactions of Q22 with LolA may contribute to the phenotypic changes seen here.

In conclusion, we have shown that sub-inhibitory concentrations of Q22 can alter and affect the phenotypic traits of certain BCC. Importantly, in contrast to earlier studies performed in vitro (unpublished data), our studies demonstrate that Q22 is highly toxic for zebrafish larvae and mice, which suggest that this compound is an unacceptable candidate for drug development for the treatment of human disease.

Furthermore, as destabilisation of the bacterial cytoskeleton using compounds such as Q22 can lead to unexpected increases of in-vitro, virulence-related traits, we believe future development of antimicrobials targeting the BCC bacterial cytoskeleton may be hampered if such effects translate into the in-vivo environment of CF infection, and consider that caution must be taken. Future studies that study a wider panel of isolates may help understand how widespread our observations are across the BCC. Further studies to help our understanding of the mechanistic basis of this increased virulence are imperative for developing alternative control strategies.

## 4. Materials and Methods

### 4.1. Ethics Statement

Mammalian studies were conducted according to protocols approved by the San Raffaele Scientific Institute (Milan, Italy) Institutional Animal Care and Use Committee (IACUC), and adhered strictly to the Italian Ministry of Health guidelines for the use and care of experimental animals. Zebrafish (Danio rerio) were kept and handled in compliance with the guidelines of the European Union for handling laboratory animals (http://ec.europa.eu/environment/chemicals/lab_animals/home_en.htm). Studies performed at VBMI are approved by the Direction Départementale de la Protection des Populations (DDPP) du Gard (ID 30-189-4). The experiments were terminated before the larvae reached the free feeding stage and did not classify as animal experiments according to the 2010/63/EU Directive. Approval date: 1 October 2014.

### 4.2. Bacterial Strains and Growth Conditions

The bacterial strains used in this study are included in the international BCC reference panel [20] and are described in Table 1. BCC strains were grown at 37 °C in Luria-Bertani (LB) broth, unless otherwise indicated. Q22, kindly provided by Prof. John Perry [8], was maintained in methanol at 1 mg/mL and stored at −20 °C. Bacterial stocks were maintained at −80 °C as 20% glycerol suspensions.

### 4.3. Q22 Treatment

Q22 treatment doses were based on sub-MIC concentrations, unless otherwise indicated [8]. To measure the effects of Q22 treatment on BCC cell morphology and growth, overnight bacterial cultures were subcultured 1:500 into fresh LB broth, in the absence or presence of Q22, and incubated at 37 °C with shaking. LB broth was supplemented with Q22 at the 0 h timepoint. Samples were removed at selected timepoints as indicated. BCC were additionally grown in the presence of corresponding volumes of methanol, as a diluent control. A viable count of bacteria in cultures grown in the presence and absence of Q22 was taken to confirm acceptable use of optical density as a measure of growth. Scanning electron microscope images of the resulting cells were taken.

### 4.4. Hydrogen Peroxide (H_2_O_2_) Protection Assay

The ability of BCC strains to survive H_2_O_2_ exposure was measured, as previously described [10]. Briefly, BCC cultures were grown and shaken at 37 °C in LB, supplemented with Q22, diluent control (methanol), or no supplementation until the late stationary phase. The optical density was determined and samples were standardised to a concentration of 1 × 10^8^ cfu. Each strain was treated with varying H_2_O_2_ concentrations (0–20 mM) and incubated with shaking at 25 °C for 30 min. Control samples received distilled H_2_O in place of the H_2_O_2_ treatment. Appropriate serial dilutions were plated in triplicate on LB agar plates. Colonies were counted after 48 h incubation, and percentage survival calculated by comparison with colony counts obtained from untreated samples.

### 4.5. Isolation of Bacterial Whole Cell Lysates (WCLs)

Whole cell lysates (WCLs) were prepared as described, with additional modifications [24]. Briefly, strains were grown overnight on Brain Heart Infusion (BHI) agar plates or in BHI broth to an optical density of ~1.8 at 600 nm. Broth grown bacteria were harvested by centrifugation and washed once with phosphate-buffered saline (PBS), to remove growth media and supplements prior to standardising to an optical density of 0.2 at 600 nm. Bacterial suspensions were disrupted by sonication with a Branson 150 sonifier (six cycles, 30 s on, 30 s off) and incubated with 200 μg/mL deoxyribonuclease II (Dnase II) (Sigma-Aldrich, Saint Louis, MO, USA) at 37 °C for 1 h. WCLs were treated with 2 mg/mL Proteinase K (Sigma-Aldrich) at 60 °C for 2 h, boiled for 20 min (inactivating Proteinase K), and stored at −80 °C until required.

### 4.6. Purification of Lipopolysaccharide (LPS)

Lipopolysaccharide (LPS) was extracted and purified using a modified version of the hot-water phenol method previously described [25]. Strains were grown to late log phase in nutrient broth containing 0.5% yeast extract at 37 °C. Bacteria were harvested by centrifugation at 1000× *g*, re-suspended in a minimal volume distilled water, and freeze-dried. Pellets were re-suspended in distilled water and sonicated on ice. The resulting sonicated suspension was subjected to Dnase II digestion (final concentration 200 μg/mL) at 37 °C for 2 h, followed by Proteinase K digestion (final concentration 1 mg/mL) at 60 °C for 2 h. After boiling for 20 min, an equal volume was mixed with hot phenol and incubated at 70 °C for 20 min with regular mixing. The suspension was cooled on ice and centrifuged at 800× *g*. The water-soluble phase was removed and dialysed against repeated changes of fresh distilled water for 72 h. Ultracentrifugation at 39,500× *g* for 6 h at 13 °C was undertaken, and the supernatant was discarded. The resulting pellet was dissolved in a minimal volume of ultrapure distilled water and freeze-dried. Protein and DNA contamination levels were assessed using a Pierce™ BCA Protein Assay (Thermo Scientific, Waltham, MA, USA) and UV spectrophotometry, respectively, to ensure an LPS purity level of at least 95% was obtained. LPS samples were analysed by sodium dodecyl sulphate polyacrylamide gel electrophoresis (SDS-PAGE) and visualised using a Pierce™ Silver Stain kit (Thermo Scientific).

After removal of organic solvents under a vacuum, the lipooligosaccharide (LOS) fraction was precipitated from concentrated phenol solution with water; the precipitate was washed with aqueous 80% phenol, and then three times with cold acetone and then lyophilized. The LOS fractions were analyzed by SDS-PAGE on 16% gels, which were stained with silver nitrate

### 4.7. Isolation of Lipid A

Free lipid A was obtained by hydrolysis of LOS (with 10 mM sodium acetate buffer pH 4.4, (100 °C, 3 h). The solution was extracted three times with chloroform/methanol/H_2_O (100:100:30 *v*/*v*/*v*) and centrifuged (4 °C, 5000× *g*, 15 min). The organic phase contained the lipid A, and the water phase contained the core oligosaccharide.

### 4.8. Matrix Assisted Laser Desorption/Ionization Time of Flight (MALDI-TOF) Mass Spectrometry

MALDI-TOF mass spectra of the intact LOS were recorded in the negative polarity on a Perseptive (Framingham, MA, USA) Voyager STR equipped with delayed extraction technology. Ions formed by a pulsed UV laser beam (nitrogen laser, λ = 337 nm) were accelerated by 24 kV.

LOS and lipid A sample preparation: R-type LOS MALDI preparation was performed as recently reported in detail [26]. MALDI preparation of lipid A was performed as described [27]. Briefly, samples were dissolved in chloroform/methanol (1:1 *v*/*v*), whereas matrix solution was prepared by dissolving 2,4,6-trihydroxyacetophenone (THAP) in methanol/0.1% trifluoroacetic acid/acetonitrile (7:2:1). A sample/matrix solution mixture (1:1 *v*/*v*) was deposited (1 μL) onto the MALDI plate and left to dry at room temperature.

### 4.9. Purification of Peptidoglycan (PG)

Sacculi were isolated from BCC as described [28], with the following modifications. BCC cells (800 mL) were grown to an optical density of 0.6 at 600 nm and harvested by centrifugation at 7500× *g* for 15 min at 4 °C. The cell pellet was re-suspended in 6 mL of ice-cold sterile dH_2_O. The cell suspension was added dropwise to 6 mL boiling 8% sodium dodecyl sulphate (SDS) solution. The solution was boiled for a further 30 min, and the resulting cell suspension pelleted by centrifugation at 130,000× *g* for 60 min at 25 °C. The pellet was washed repeatedly with dH_2_O until the supernatant was free of SDS, as determined by a published assay [29].

### 4.10. Cell Culture

THP-1 human monocytes [30] were kindly donated by Prof. John Taylor and were maintained at 37 °C with 5% CO_2_ in RPMI-1640 medium (Sigma-Aldrich), supplemented with 10% foetal calf serum (Sigma-Aldrich), 1% penicillin/streptomycin (Sigma-Aldrich) and 1% l-glutamine (Sigma-Aldrich). To differentiate the monocytes into macrophage-like cells, 300 μL cells at a concentration of 0.5 × 10^6^ cells/mL was transferred into 24-well tissue culture plates and incubated at 37 °C with 5% CO_2_ for 24 h in the presence of 2.5 ng/mL phorbol myristate acetate (PMA) (Sigma-Aldrich). Prior to stimulation, the differentiated cells were washed with PBS.

### 4.11. Stimulation Assays and Cytokine Quantification

Differentiated THP-1 cell lines were stimulated for 6 h with either 12.5 μL/mL bacterial whole cell lysate, 100 ng/mL LPS, or 100 µg/mL PG as indicated. Culture supernatants were harvested and assayed for cytokine (TNFα and IL-1β) production using Human DuoSet^®^ ELISA Kits (R&D Systems, Abingdon-On-Thames, UK). Where indicated, differentiated THP-1 cells were pre-incubated with CD14 antibody (Monosan, Uden, The Netherlands, Mon1108, final concentration 400 ng/mL) for 1 h prior to stimulation.

### 4.12. Zebrafish Model

To determine the toxicity of compound Q22 in vivo, zebrafish embryos staged 30–32 h post-fertilisation (hpf) were incubated with E3 embryo medium (5 mM NaCl, 0.17 mM KCl, 0.33 mM CaCl_2_, 0.33 mM MgSO_4_) containing increasing concentrations (0, 6.4 µg/mL, 64 µg/mL or 640 µg/mL) of Q22. A stock solution of 6.4 mg/mL was freshly prepared in E3. Embryos, individually plated in a 24-well plate (experiment 1: *n* = 10 per treatment), or in groups of 10–20 embryos in 6-well plates (experiment 2: *n* = 40 per treatment; experiment 3: *n* = 50 per treatment) were incubated at 29 °C. Embryos were observed for five days post-administration, and embryo death was determined by the absence of a heartbeat.

### 4.13. Mouse Model

To determine the toxicity of compound Q22 in mammals, 8–10 week old male C57BL/6NCr mice, 20–22 g, from Charles River Laboratories, were housed in filtered cages under specific pathogen-free conditions, and permitted unlimited access to food and water. Groups of two mice were inoculated via the intratracheal (IT) route with varying doses (100 mg/kg; 50 mg/kg; 25 mg/kg) of Q22 dissolved in 60 µL distilled dH_2_O. Control animals were exposed to 60 µL dH_2_O as a negative control. For IT injection, mice were anesthetized by an intraperitoneal injection of Avertin (2,2,2-tribromethanol, 97%) in 0.9% NaCl, administered at a volume of 0.015 mL/g body weight. Mice were placed in a supine position. The trachea was directly visualised by ventral midline, exposed and intubated with a sterile, flexible 22 g cannula attached to a 1 mL syringe. After compound administration, moribund mice (those that could not right themselves after being placed in lateral recumbence) were killed before termination of the experiments. Remaining groups of mice were monitored twice daily for the parameters: vocalisation, piloerection, attitude, locomotion, breathing, curiosity, nasal secretion, grooming, and dehydration. Mice that lost >25% of their body weight and had evidence of severe clinical disease, such as scruffy coat, inactivity, loss of appetite, poor locomotion, or painful posture, were sacrificed before the termination of the experiments with an overdose of carbon dioxide. Mice were sacrificed by increasing CO_2_ administration, and lungs were harvested and observed for damage and inflammation.

### 4.14. Statistical Methods

Statistical analysis was performed using a paired *t*-test where appropriate. Differences with *p* values of <0.05 were considered statistically significant. For zebrafish survival studies, the data were plotted using Kaplan–Meier survival curves (Prism GraphPad software version 6.03), and statistical significance was determined with a log rank test.

## Figures and Tables

**Table 1:** Bacterial strains used in this study.

Species	Description	Source
*B. cenocepacia* LMG16656 (J2315)	Clinical isolate, CF patient, ET-12 epidemic strain	BCCM
*B. cenocepacia* LMG18829	Clinical isolate, CF patient, epidemic strain	BCCM
*B. multivorans* LMG13010	Clinical isolate, CF patient	BCCM
*B. vietnamiensis* LMG16232	Clinical isolate, CF patient	BCCM

BCCM, Belgian Coordinated Collection of Micro-organisms.

**Table 2:** Structural analysis of *B. cenocepacia* LMG18829 lipid A.

	*B. cenocepacia* LMG18829 Untreated		*B. cenocepacia* LMG18829 + 30 µg/mL Q22	
Lipid A Species	Intensity (%)	Mass	Intensity (%)	Mass
tetra-acylated lipid A			58.8	1444.9
tetra-acylated lipid A + Ara4N	100	1576.4	100	1576.1
tetra-acylated lipid A + 2 Ara4N	87.8	1707.5	60	1707.0
penta-acylated lipid A + Ara4N	48.7	1800.9	38.2	1801.0
penta-acylated lipid A + 2 Ara4N	39	1934.0	29	1932.2

Negative ion MALDI-TOF spectrum analysis of lipid A isolated from *B. cenocepacia* LMG18829, grown in the presence and absence of 30 µg/mL Q22.

**Table 3:** Toxicity of Q22 in the mouse model.

Group	Dose Q22 (mg/kg)	Results
1	100	Mice died immediately after intra-tracheal administration of Q22
2	50	Mice died immediately after intra-tracheal administration of Q22
3	25	Mice showed reduced mobility compared with controls during first 2 days after treatment. Lungs were inflamed and damaged after 4 days
4	0	Mice were healthy after treatment

Mice were injected IT with increasing concentrations of Q22 (25 mg/kg; 50 mg/kg; 100 mg/kg) and monitored for up to 4 days post administration.
